# Frequency and Impacts of Verbal Abuse on Healthcare Workers in a Secondary Healthcare Structure in Greece

**DOI:** 10.7759/cureus.35406

**Published:** 2023-02-24

**Authors:** Aikaterini Toska, Maria Saridi, Anastasios Christakis, Sofia Gotsi, Evangelos C Fradelos, Georgia Papageorgiou, Kyriakos Souliotis

**Affiliations:** 1 Department of Nursing, University of Thessaly, Larissa, GRC; 2 Department of Social & Educational Policy, University of Peloponnese, Corinth, GRC; 3 Department of General Surgery, General Hospital of Corinth, Corinth, GRC; 4 Department of Research, Health Policy Institute, Athens, GRC

**Keywords:** emotional reactions, violence, impact, work envirroment, physicians, nurses, verbal violence, incidence, healthcare workers, verbal abuse

## Abstract

Background: Threatening and aggressive behaviors in healthcare settings constitute a significant problem that can affect not only the physical and mental integrity of staff but also patient safety and the quality of healthcare. Literature highlights verbal abuse as the most common form of non-physical violence and is estimated from 51.8% to 63.4% worldwide.

Purpose: The purpose of the study was the investigation of verbal abuse incidence toward physicians and nurses and the differences noted between them in a regional hospital.

Methods: The study took place in a public regional hospital in southern Greece. A number of 185 nurses and 60 physicians completed the verbal abuse scale (VAS) for assessing verbal violence in the work environment; selected socio-demographic and occupational characteristics were also recorded.

Results: Physicians have undergone verbal abuse once a week at a percentage of 38.3%, while at the same frequency; nurses record a percentage of 12.4%. Regarding the relationship between the victim of verbal abuse and the abuser, 26.7% of the physicians answered that the abuser was another senior member of the staff, while the percentage for nurses is higher and reaches 31.9%. According to VAS items, accusing and blaming (mean±SD=3.00±0.96) is noted as the most common form of verbal abuse for physicians, judging and criticizing stands out as the most frequent (3.17±1.11) and the most stressful action (3.25±1.11) form for nurses. The most frequent behavior by the physicians is to put the verbal abuse situation in a humorous context (2.78±1.14). In contrast, nurses are trying to clarify any misunderstanding that may occur (3.10±1.00).

Conclusions: Verbal abuse incidents are experienced by both physicians and nurses. They are stressful for the victims and can significantly affect work relationships and job satisfaction.

## Introduction

Workplace violence (WPV) is defined as “incidents where staff is abused, threatened, or assaulted in work-related circumstances, including commuting to and from work, involving an explicit or implicit challenge to the safety, well-being or their health" [1]. Aggressive behaviors range from verbal to physical assaults and can occur in every work environment, with health care documented as the most common workplace conflict involving approximately 62% of workers in the health sector [2]. Threatening and aggressive behaviors in health care settings are a significant problem that can impact not only the physical and mental integrity of staff but also patient safety and the quality of health care [3,4].

Violence is not necessarily physical; it can be verbal, psychological, or even sexual, with the last concerning mainly female health workers. Sometimes, emotional or verbal attacks can have much more severe effects than physical abuse, such as increasing frustration and despair [4]. Several international studies confirm the frequent exposure of health professionals to violence, with verbal abuse being the most common form of non-physical violence [2,5], reaching an average of 2.29 episodes of verbal aggression per eight hours [6], followed by threats and sexual harassment [2,6].

Even though verbal assault is the most common form of violence in healthcare settings, this type of incident is not considered worthy of reporting, resulting in a significant proportion of underestimated and unreported [7]. Given the limited data on the magnitude of the problem, the purpose of the study was to assess the frequency of sources of abusive behavior and the impact of verbal abuse incidents on physicians and nurses in a public regional hospital in Greece.

## Materials and methods

Sample

The study took place in a public regional hospital in southern Greece. A total of 200 nurses and 75 doctors worked in the hospital during the collection period; all of them were invited to participate after being informed about the purpose of the study. The final survey sample was 185 nurses (meaning a response rate of 92.5%) and 60 physicians (a response rate of 80%); since participation was voluntary, the convenience sampling was chosen. To be ensured confidentiality, the method of anonymous self-completion by healthcare professionals was followed.

Study questionnaire

The research tool consisted of two sections. The first section contains elements to record socio-demographic (sex, age, and educational level) and occupational characteristics (job role and years of experience in hospitals), while the second section includes a translated and validated Greek verbal abuse scale (VAS) version for assessing verbal violence in the work environment. The Greek version of VAS was developed in 2015 [8] and indicates high internal consistency (Cronbach's alpha=0.98). The scale consists of 11 groups of questions forming a total of 79 questions in five subscales, which are referring to how often and how intensely an employee receives verbal violence, how he reacts to it, how he manages it, and how he feels about it.

Statistical analysis

All data were analyzed using SPSS version 25 (IBM SPSS Statistics for Windows, Version 25.0., Armonk, NY, USA). Data are presented as descriptive statistics (frequency, percentage distribution, mean, and SD), while the comparison was carried out between physicians and nurses. A t-test using independent samples was used to determine whether there is a significant relationship between these two groups. The level of statistical significance was set at 0.05.

Ethics

Relevant permission to carry out the study was requested and obtained by the hospital administrations involved (Ethics Committee of General Hospital (GH) of Corinth, Protocol no. 25776/30-10-20). Anonymity and confidentiality were maintained in all stages of the survey; moreover, the participants provided informed consent to participate in the study.

## Results

Socio-demographic and occupational characteristics

The sample consists of 245 health professionals, of which 185 are nurses and 60 are physicians. Table 1 presents the socio-demographic and occupational characteristics divided into two different groups, physicians and nurses. Most physicians are male, aged less than 40 years old, and holding a postgraduate degree. In contrast, most nurses are females, aged more than 40 years old, with a university or technological institute degree and less than 10 years of experience in a hospital.

**Table 1:** Socio-demographic and occupational characteristics of the sample. *Mean (SD): For the continuous variable of age, mean and standard deviation are used instead of frequency and percentage of the total.

Characteristics	N (%)	
	Physicians	Nurses
Gender		
Male	37 (61.7%)	34 (18.4%)
Female	23 (38.3%)	151 (81.6%)
Age	39.7 (11.02)*	40.9 (9.44)*
≤40 Years old	31 (51.5%)	90 (48.6%)
>41 Years old	29 (48.5%)	95 (51.4%)
Education level		
Secondary	0	8 (4.3%)
Technological	0	64 (34.6%)
University	25 (41.7%)	65 (35.1%)
Postgraduate	30 (50%)	46 (24.9%)
Doctorate	5 (8.3%)	2 (1.1%)
Years of experience in a hospital		
≤10 Years	30 (50%)	97 (52.4%)
>10 Years	30 (50%)	88 (47.6%)

Elements of verbal abuse

Most physicians and nurses answered that verbal abuse occurred in the presence of other people (71.7% and 81.6%, respectively). The frequency of verbal abuse between professionals in recent years as well as their opinion on the frequency with which nurses experience verbal abuse are presented in Table 2. Specifically, 38.3% of physicians stated that they underwent verbal abuse once a week, while in the same frequency, nurses recorded a percentage of 12.4%. On the other hand, 28.3% of physicians believe that nurses have been subjected to verbal abuse once a week, and at the same time, only 18.9% of nurses believe the same about their colleagues.

**Table 2:** Frequency of verbal abuse (for themselves and their opinion of nurses).

Verbal abuse occurrence	Frequency of experiencing verbal abuse in recent years (answering for themselves)		Frequency of nurses experiencing verbal abuse in recent years	
	Physicians	Nurses	Physicians	Nurses
	N (%)	N (%)	N (%)	N (%)
Never	1 (1.7%)	3 (1.6%)	1 (1.7%)	5 (2.7%)
Once a year	5 (8.3%)	50 (27%)	7 (11.7%)	39 (21.1%)
Several times a year	11 (18.3%)	49 (26.5%)	13 (21.7%)	24 (13%)
Once a month	10 (16.7%)	27 (14.6%)	9 (15%)	22 (11.9%)
Once a week	23 (38.3%)	23 (12.4%)	17 (28.3%)	35 (18.9%)
Several times a week	7 (11.7%)	20 (10.8%)	10 (16.7%)	25 (13.5%)
Every day	3 (5%)	13 (7%)	3 (5%)	35 (18.9%)

**Figure 1:**
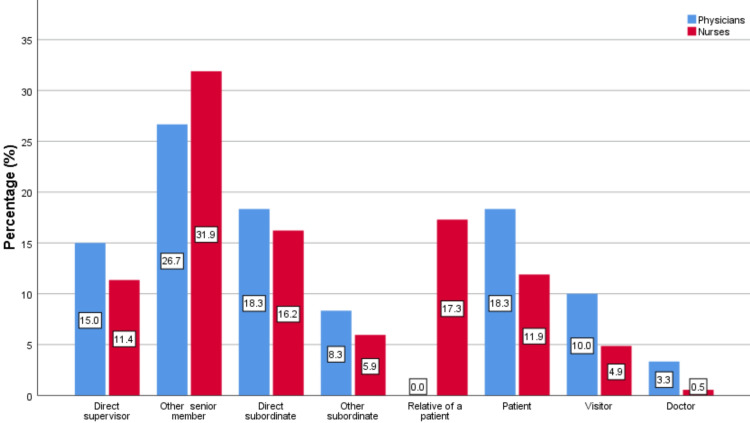
Relationship between the victim of verbal abuse and the abuser.

**Figure 2:**
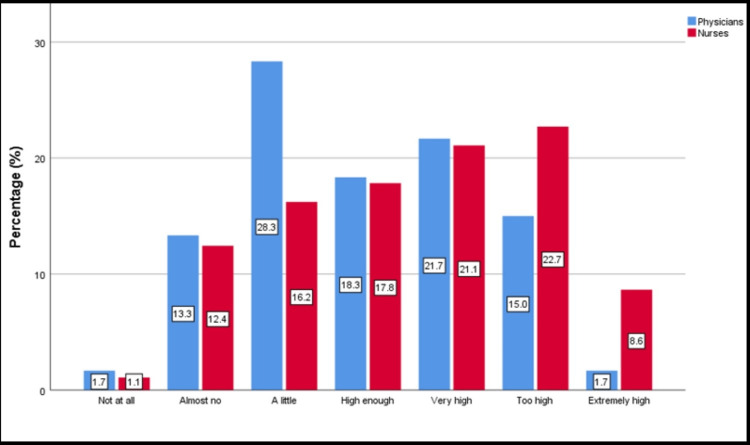
Level of stress resulting from verbal abuse.

In addition, 56.7% of physicians and 31.9% of nurses consider that the abuser didn’t realize the results of their abusive behavior. Concerning the relationship between the victim of verbal abuse and the abuser, 26.7% of the physicians answered that the abuser was another senior member of the staff, while the same percentage for nurses is higher and reaches 31.9% (Figure 1). Furthermore, 31.3% of nurses stated too high or extremely high-stress levels resulting from verbal abuse, while the same percentage for physicians reaches 16.7% (Figure 2).

Frequency and intensity of verbal abuse forms

Frequency and intensity of verbal abuse forms and the significance of each form between physicians and nurses are presented in Table 3. Accusing and blaming (3.00±0.96) is the most common form of verbal abuse for physicians and the most stressful action too (2.87±1.02). Judging and criticizing stands out as the most frequent (3.17±1.11) and the most stressful action (3.25±1.11) forms for nurses. Moreover, statistically significant differences were found between the two health professionals' categories. Concerning frequency, nurses are more likely to experience verbal abuse than physicians through the forms of abusive anger (p=0.049), abuse disguised as a joke (p=0.024), and judging and criticizing (p=0.006). Furthermore, nurses experienced verbal abuse in greater intensity; through the forms of trivializing (p=0.002), judging and criticizing (p=0.000), threatening (p=0.002), and sexual harassment (p=0.000).

**Table 3:** Frequency and intensity of verbal abuse forms between physicians and nurses (mean, standard deviation, and significance between groups).

Verbal abuse forms	Frequency		P-value	Stressfulness		P-value
	Physicians	Nurses		Physicians	Nurses	
Abusive anger	2.75±0.88	3.04±1.14	0.043	2.83±0.92	3.11±1.17	0.059
Condescending	2.65±0.73	2.84±3.14	0.637	2.55±0.65	2.61±0.97	0.616
Abuse disguised as a joke	2.63±0.88	3.00±1.15	0.024	2.72±0.88	2.92±1.11	0.187
Ignoring	2.53±0.81	2.68±1.15	0.282	2.67±0.86	2.85±1.16	0.196
Trivializing	2.62±0.88	2.88±1.17	0.109	2.68±0.91	3.15±1.14	0.002
Accusing and blaming	3.00±0.96	2.96±1.19	0.776	2.87±1.02	3.14±1.22	0.093
Judging and criticizing	2.72±0.99	3.17±1.11	0.006	2.68±0.95	3.25±1.11	0.000
Discounting	2.73±0.95	2.84±1.14	0.501	2.65±0.94	2.89±1.10	0.128
Threatening	2.55±0.96	2.76±1.23	0.174	2.53±1.00	3.10±1.24	0.002
Sexual harassment	2.02±0.87	2.26±1.16	0.089	1.77±0.81	2.55±1.32	0.000

First thoughts after the verbal abuse incidence

The first thought after the incidence of verbal abuse mentioned more often by physicians is “What a jerk” (3.05±0.91), while the one mentioned by nurses is “He/she has no right to treat me this way.” Nurses were more likely to think that they didn't deserve to be treated this way (p=0.002), that the abuser had no right to treat them this way (p=0.000), and that they hadn’t done anything wrong (p=0.001) than physicians (Table 4).

**Table 4:** First thoughts after the verbal abuse incidence (mean, standard deviation, and significance between groups).

First thoughts	Physicians	Nurses	P-value
What a jerk	3.05±0.91	2.97±1.28	0.584
I don't deserve to be treated this way	2.93±0.99	3.42±1.17	0.002
Ι can deal with this	2.80±0.95	2.94±1.09	0.372
He/she has no right to treat me this way	2.80±1.04	3.57±1.19	0.000
I haven't done anything wrong	2.70±0.94	3.21±1.23	0.001
I am going to be in trouble over this	2.48±1.00	2.46±1.12	0.883
Why can't I do something right when I work with him/her	2.38±0.90	2.48±1.15	0.499
It doesn't matter	2.53±0.93	2.49±1.04	0.756
I can't handle this	2.50±0.95	2.39±1.02	0.479
This could potentially hurt me	2.38±0.86	2.65±1.09	0.088
It must be my fault	2.38±0.92	2.24±1.04	0.341
I have never had a problem with anyone else	2.60±1.03	2.58±1.11	0.921
Why am I always the one who causes him/her a problem? He/she is not shouting at anyone else	2.42±0.94	2.18±1.04	0.124
Why do I let him/her upset me enough to cry?	2.22±1.06	2.28±1.04	0.679

Coping behaviors and their perceived effectiveness

Coping behaviors refer to the reaction of the health professional after the verbal abuse they received (Table 5). The most frequent behavior by the physicians is to put the verbal abuse situation in a humorous context (2.78±1.14), while the nurses try to clarify any misunderstandings that may occur (3.10±1.00). According to statistical tests, nurses are more likely than physicians to attempt to clarify misunderstandings (p=0.000), engage in positive activities that directly reduce their tension (p=0.002), talk to themselves in a reassuring way (p=0.000), deal directly with the abuser about the abuse (p=0.011), and detach themselves from the situation (p=0.004).

**Table 5:** Coping behaviors and their perceived effectiveness (mean, standard deviation, and significance between groups).

Coping behavior	Physicians	Nurses	P-value
I try to put the situation in perspective	2.62±0.88	2.79±1.02	0.226
I ask for assistance/support from others	2.33±0.79	2.57±1.01	0.061
I attempt to clarify any misunderstanding	2.43±0.83	3.10±1.00	0.000
I engage in positive activities that directly reduce my tension	2.47±0.77	2.92±1.03	0.002
I talk to myself in a reassuring way	2.50±0.89	3.09±1.16	0.000
I deal directly with the abuser about the abuse	2.42±0.89	2.78±1.16	0.011
I become silent with the abuser	2.42±0.89	2.57±1.17	0.322
I detach myself from the situation	2.45±0.87	2.88±1.03	0.004
I try to keep my feelings to myself	2.58±0.96	2.77±1.24	0.241
I engage in controversial activities that reduce my tension	2.38±0.82	2.64±0.96	0.069
I tend to blame myself	2.38±0.82	2.15±0.98	0.100
I engage in wishful thinking	2.52±0.93	2.70±1.02	0.212
I try to deal with humor	2.78±1.14	2.69±1.09	0.576

Effects of verbal abuse

The effects obtained after verbal abuse by health professionals are presented in Table 6. Both physicians and nurses display the most often effect on the deterioration of the relationship with the colleague (2.38±1.01 and 2.60±1.06, respectively). Additionally, significant differences were recorded in several effects: decreased trust and support in the workplace (p=0.010), impact on mental health (p=0.000), reduced job satisfaction (p=0.019), and protest of other employees as they shoulder the abstention of the verbally abused colleague (p=0.004). Nurses noted higher scores compared to physicians in all of them.

**Table 6:** Effects of verbal abuse (mean, standard deviation, and significance between groups).

Effects	Physicians	Nurses	P-value
Deterioration of the relationship with the colleague	2.38±1.01	2.60±1.06	0.165
Deteriorating relationships with people outside the work environment	2.15±0.97	2.22±0.97	0.620
Deterioration of the relationship with the rest of the staff	2.10±0.97	2.20±0.99	0.494
Decreased trust and support in the workplace	2.20±0.90	2.58±1.02	0.010
Decreased self-esteem	2.05±0.89	2.16±1.11	0.406
Decreased self-confidence	2.13±0.91	2.09±1.04	0.782
Impact on mental health	1.98±0.91	2.51±1.06	0.000
Reduced performance and compliance with professional requirements	2.08±1.00	2.17±1.00	0.547
Health burden	2.08±0.94	2.36±1.01	0.060
Reduce the feeling of comfort/well-being in the workplace	2.27±0.95	2.49±0.99	0.124
Reduced job satisfaction	2.13±0.96	2.50±1.06	0.019
Increase of hours of absence from work	1.93±0.88	1.93±0.90	0.989
Protest of other employees as they shoulder the abstention of the verbally abused colleague	1.93±1.04	2.39±1.07	0.004

Emotional reactions after verbal abuse incidence

Emotional reactions of health professionals after the verbal abuse are presented in Table 7. The most common emotional reaction of physicians is to feel frustrated (3.12±0.94), and for nurses, it is anger (3.16±1.09). Significant differences emerged in some emotional reactions. Specifically, disgust (p=0.025) and discomposure (p=0.018) are more often felt by nurses, and on the contrary, physicians are more likely to feel responsible (p=0.047) or defeated (p=0.043).

**Table 7:** Emotional reactions after verbal abuse incidence (mean, standard deviation, and significance between groups).

Emotional reaction	Physicians	Nurses	P-value
Frustration	3.12±0.94	3.03±1.01	0.569
Anger	2.93±0.92	3.16±1.09	0.112
Disgust	2.80±0.97	3.15±1.07	0.025
Embarrassment or humiliation	2.62±0.88	2.67±1.06	0.733
Sadness	2.82±0.98	3.03±1.03	0.154
Helpless	2.52±0.96	2.31±1.06	0.190
Powerless	2.53±0.95	2.32±1.10	0.179
Sock and surprise	2.78±0.92	2.80±1.12	0.890
Confused	2.66±0.96	2.50±1.03	0.306
Responsible	2.60±1.03	2.28±1.09	0.047
Threatened	2.78±0.98	2.65±1.16	0.398
Discomposure	2.50±0.85	2.83±1.14	0.018
Defeated	2.47±0.98	2.17±0.99	0.043
Indifferent	2.62±0.99	2.42±1.02	0.198
Feared	2.33±0.86	2.32±0.96	0.949
Isolated	2.33±0.88	2.25±0.95	0.576
Misunderstood	2.60±0.94	2.65±1.01	0.742
Not supported	2.55±1.02	2.45±1.06	0.538

## Discussion

The phenomenon of verbal abuse is usually more prevalent in healthcare settings compared to physical attacks [9], but it is still more difficult to assess due to the under-reporting of these incidents [7]. Given the high incidence of this form of abuse in healthcare settings [10,11], the purpose of the present study was to investigate the prevalence of verbal abuse among physicians and nurses in all departments of a general hospital in Greece. According to our results, almost four out of ten physicians reported they experienced verbal violence at least once a week, while only one out of ten nurses said this happens with the same frequency. This finding contrasts with a similar survey conducted in Greek hospitals, which showed that verbal abuse among nursing staff occurred several times a week [12,13]. Studies in hospital operating rooms also showed that nurses are more often victims of verbal, psychological, or physical abuse compared to physicians, demonstrating that the operating room is an environment of frequent confrontation and increasing tension that raises the likelihood of violent episodes [10,11,14].

Regarding the relationship between the victim of verbal abuse and the abuser, almost the same proportion of both professional categories reported a senior member as the abuser. Other research showed different sources of violence for physicians, such as patients or attendants [15-19], while for nurses, the researchers agree that the most common sources of verbal abuse are physicians, who often release their tension and stress on their colleagues [8,12-14], as well as patients and attendants [19,20-23], which are more likely to inflict physical violence on nursing staff [24], often holding them responsible for the quality of care provided. The origin of violence from different sources highlights the need to improve and effectively manage violent incidents in the healthcare environment.

Concerning the form of verbal violence, the most frequent form of abuse is accusing/blaming physicians and criticism of nurses, a finding that agrees with that of other studies [14,25]. Nurses in our study also experienced abusive anger, abuse disguised as a joke, and belittling with greater frequency than physicians. Another study found that the most frequent and stressful types of verbal abuse experienced by preoperative nurses were abusive angry behaviors [26]. Regarding thoughts and feelings after the incident, it seems that nurses experience twice as high levels of stress compared to physicians. Nurses also considered to a greater extent that the abusers carry out the attack having realized the results of their abusive behavior.

A difference was also observed in the reactions between physicians and nurses after the incident of verbal violence; physicians are mainly possessed by frustration and a sense of defeat, while nurses feel anger and a sense of disgust. Anger and feelings of disgust were common for physicians and nurses in a similar research study on the phenomenon of verbal violence in operating theaters of three hospitals in Greece [14]. Numerous international studies agree that anger is the most common normal emotional response after an episode of verbal abuse and is often associated with negative thoughts and actions [27,28], while other studies have shown that victims of verbal abuse, in addition to being angry, often felt surprised and shocked [26,28]. Regarding the first thoughts after the incident, there was also a difference between nurses and physicians. The latter tend to belittle the abuser by characterizing him as a “jerk,” placing the verbal abuse in a humorous context without dealing with the incident further, while nurses think that the abuser has no right to behave in this way, trying to clarify any misunderstanding or even removing themselves from the situation.

In similar studies, the thought that the abuser has no right to act against this type of behavior also concerns physicians, causing significant damage of violence to the self-esteem and self-respect of the victims, who try to solve the possible misunderstanding [14,29]. In other studies, a positive approach of the victims to managing verbal violence is reported, using work experience and the ability to adapt to difficult conditions and tension in the work environment [25,27]. The deterioration of work relationships was mentioned as the main result of verbal violence, with the most important being the loss of trust on the part of nurses, the impact on their mental health, but also reduced job satisfaction. The effect of the verbal episode on work relationships is a finding of another study [14] demonstrating that verbal violence significantly affects the quality of care due to the turmoil it creates and the disruption of the cohesion of the workgroup, leading to reduced performance, increased absenteeism rates, and even leaving the workplace [25,28].

## Conclusions

Both physicians and nurses have equally been victims of verbal abuse incidents in the work environment. The main types of violence experienced by professionals were accusations and criticism, which caused different emotions and triggered different reactions among healthcare professionals, with nurses being angrier and more disgusted, choosing a more dialectical approach than physicians, who felt more frustrated and defeated and tended to underestimate the abuser. It was documented that verbal violence can affect work relationships, deteriorates the work environment, and has an impact on the victims’ mental health, job satisfaction, and the quality of work performance. A better understanding of this phenomenon is needed, in order for the deeper causes of verbal abuse to be clarified. Psychological support, empowerment and training for the management of such events should be a priority for all healthcare settings.
